# Rapunzel syndrome with cholangitis and pancreatitis – A rare case report

**DOI:** 10.1515/med-2020-0243

**Published:** 2020-11-14

**Authors:** Rajendran Vellaisamy, Shruti Iyer, Servarayan Murugesan Chandramohan, Sakthivel Harikrishnan

**Affiliations:** Department of General Surgery, Madras Medical College, EsoIndia Research Volunteer, Chennai 600003, India; Sri Ramachandra Institute of Higher Education and Research, EsoIndia Research Volunteer, No. 1, Ramachandra Nagar, Porur, Chennai 600116, India; International Students Programme, Sri Ramachandra Institute of Higher Education and Research, President and Founder – EsoIndia, Chennai 600116, India; MCh Surgical Gastroenterology Resident, Department of Surgical Gastroenterology & Liver Transplant, Government Stanley Medical College EsoIndia Research Volunteer, Chennai 600001, India

**Keywords:** trichobezoar, intestinal obstruction, laparotomy, cholangitis, pancreatitis

## Abstract

Rapunzel syndrome, defined by the presence of a trichobezoar extending from the stomach to the small intestine, is a rare cause of intestinal obstruction. It usually presents with vague symptoms; however, it can also present with complications such as perforation, peritonitis and obstructive jaundice. We report a rare case of a 37-year-old woman with Rapunzel syndrome complicated by acute cholangitis and pancreatitis and analyse the diagnosis and management of this complicated pathology. Although she reported a history of trichotillomania and trichophagia, she had been asymptomatic for ten years. We review the steps of diagnosis, highlighting the importance of a thorough clinical history and detailed examination, with supporting evidence from the contrast-enhanced computed tomography (CECT) scan. She was successfully managed with gastrotomy and trichobezoar removal. She had an uneventful postoperative recovery and was discharged after psychiatric counselling. To our knowledge, this is the first case of Rapunzel syndrome in a young female presenting with both cholangitis and pancreatitis.

## 1 Introduction

Rapunzel syndrome is a rare entity defined by the presence of a trichobezoar that extends from the stomach to the small intestine. Named after the fairy-tale Rapunzel, where the titular character has long hair, the first case was described in 1968 [1]. Since then, there have been around 110 cases reported. Bezoars are concretions in the gastrointestinal tract, which increase in size as they accumulate more nonabsorbable fibres. The most common type of bezoar seen mostly in females in the paediatric age group is a trichobezoar, which is a concretion of hair from external sources that amalgamate over the years. It is usually associated with psychiatric conditions such as trichotillomania, trichophagia and pica [2].

Patients are generally asymptomatic until the bezoar becomes very large and causes intestinal obstruction. Over 70% of the cases are females younger than 20 years and present with nausea, vomiting, pain and abdominal distension [3]. Very rarely, it presents with complications such as gastric perforation (10%), intussusception (1.8%) or cholangitis (<0.9%) [4]. We present a case of Rapunzel syndrome with both pancreatitis and cholangitis, seen in a young female. To the best of our knowledge, this is the first case of Rapunzel syndrome with both complications of cholangitis and pancreatitis.

## 2 Case report

A 37-year-old female patient presented to us with complaints of continuous, dull abdominal pain for six months. She reported an acute exacerbation of the epigastric pain with radiation to the back for five days. She had a history of fever for the past five days with a maximal temperature of 101°F, for which she was treated with acetaminophen tablets (500 mg). Additional complaints included halitosis and early satiety. Ten years ago, she was diagnosed with trichotillomania with anxiety and trichophagia. She reported receiving behavioural therapy and medication (sertraline 50 mg/day) for about one year, following which she had no recurrence of symptoms and was not on any medication. She had no history of pica or any eating disorders.

The height and weight of the patient were 162 cm and 54 kg, respectively, and the body mass index was 20.6. On clinical examination, she had icterus. Abdominal examination revealed a mobile mass in the epigastrium extending to the right hypochondrium with visible gastric peristalsis. Abdominal ultrasound showed a large heteroechoic intragastric mass. Upper gastrointestinal endoscopy revealed that her entire stomach was filled with hair and the scope could not be negotiated into the duodenum. Laboratory investigations revealed increased bilirubin levels (5.2 mg/dL) and elevated total leukocyte count (17,200 cells per μL). The lipase (550 IU/L; normal – 0–160 IU/L) and amylase (450 IU/L; normal – 30–110 IU/L) levels were increased. The AST and ALT levels were 53 U/L (normal –  5–40 U/L) and 58 U/L (7–50 U/L), respectively. The ALP level was 158 U/L (normal 45–115 U/L), and the GGT level was 150 U/L (normal 9–48 U/L). ESR was also elevated at 50 mm/h, and the CRP levels were not evaluated.

**Figure 1:**
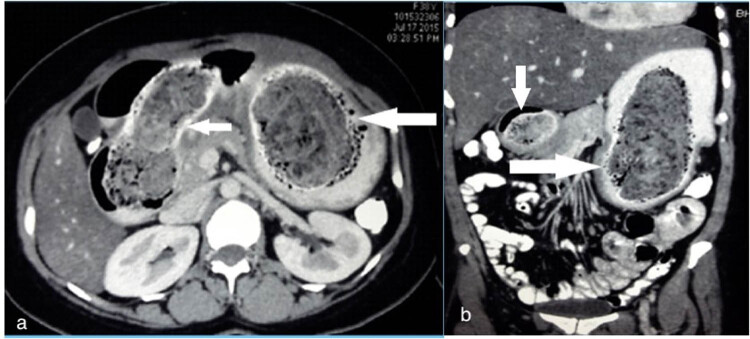
Axial (a) and coronal (b) CT scan image showing a non-homogenous non-enhancing mass with a mottled appearance within the lumen of the stomach (large arrow) first and second part of duodenum (small arrow).

The contrast-enhanced computed tomography (CECT) scan revealed an intraluminal mottled mass of mesh-like appearance occupying the entire stomach and extending beyond the second part of the duodenum, delineated by the oral contrast circumferentially, suggestive of a trichobezoar (Figure 1). The lack of any findings of biliary dilation or the presence of gallstones on the ultrasound further narrowed down the diagnosis.

Hence, due to evidence on the CT scan, a preoperative diagnosis of a trichobezoar was made. The large size and high density of the bezoar made fragmentation and removal by endoscopy, a high-risk procedure with high chances of failure. Failure of endoscopy could cause fragmentation and dislocation of parts of the bezoar. Written informed consent was obtained from the patient to perform the surgery, after a clear presentation of all available treatment options.

**Figure 2:**
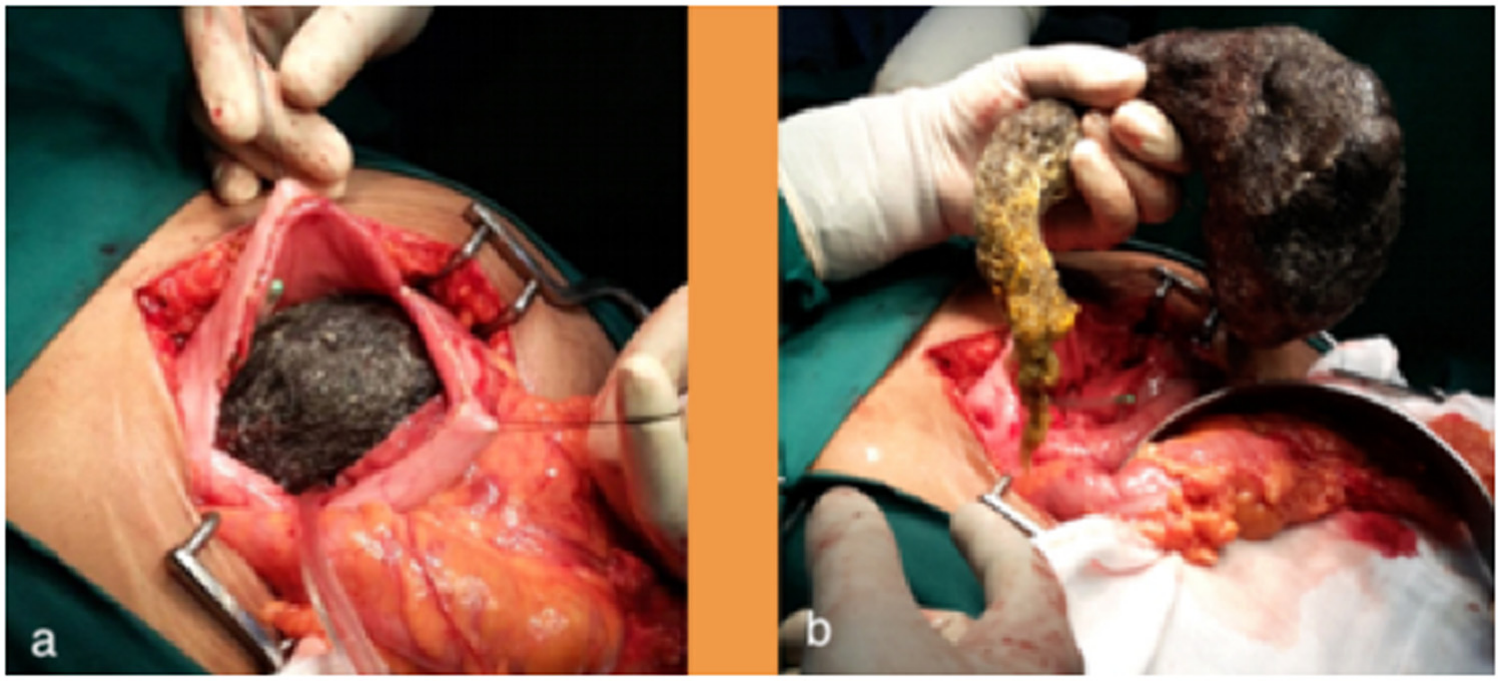
Panels (a and b) showing anterior gastrotomy and removal of the large trichobezoar extending from the stomach to the second part of duodenum

**Figure 3:**
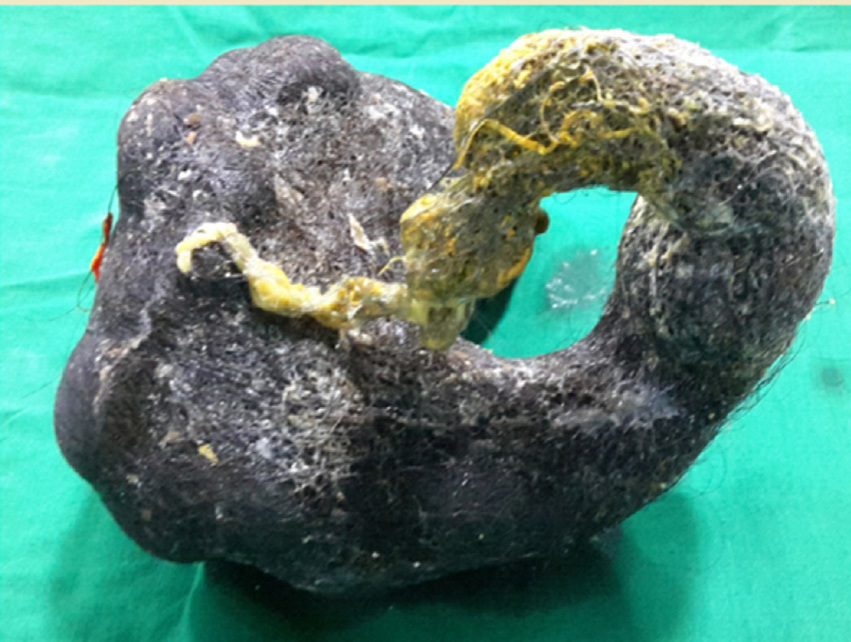
Specimen showing the removed trichobezoar casted in the shape of the stomach and the duodenum.

Given the large size of the trichobezoar and the presence of cholangitis and pancreatitis, the patient was scheduled for surgery. The abdomen was entered by a midline incision. The anterior gastrotomy was done between stay sutures which revealed a large, entangled mass of hair in the stomach with the tail extending to the second part of the duodenum (Figure 2). The tail of the trichobezoar was found to be obstructing the Ampulla of Vater, which confirmed the diagnosis of Rapunzel syndrome with complications of acute cholangitis and pancreatitis (Table 1). The trichobezoar was removed in-toto without peritoneal contamination (Figure 3). She had an uneventful postoperative recovery and was discharged after detailed psychiatric counselling. She was also advised regular follow-up, and three years later, the patient reports no complications. At the three years of follow-up (2019), informed consent was obtained from the patient for this study.

**Table 1:** Review of Rapunzel Syndrome cases complicated by pancreatitis and biliary obstruction

S. no.	Study	Age of patient	Presenting features	Complications	Management	Status
1	Jones et al. [19]	37-year-old female	Abdominal pain, loss of appetite, constipation	Small bowel obstruction Pancreatitis	Laparotomy	Alive
2	Jalali et al. [20]	17-year-old female	Intermittent bilious vomiting, mid-epigastric to right upper quadrant abdominal pain, weight loss	Esophagitis, gastritis, mild pancreatitis	Laparoscopic gastrotomy	Alive
3	Salem et al. [21]	22-year-old female	Epigastric pain, vomiting, recent history of vaginal delivery	Pancreatitis	Emergency laparotomy	Alive
4	Kohler et al. [22]	16-year-old male	Severe abdominal pain with radiation to back	Pancreatitis	Laparotomy	Alive
5	Dayasiri et al. [10]	14-year-old female	Fever, abdominal pain, vomiting worsening on eating	Acute pancreatitis and hypoalbuminemia	Laparotomy with combined gastrotomy and enterotomy	Alive
6	Hamilton et al. [23]	12-year-old female	Fatigue, nausea, decreased appetite, abdominal pain, vomiting, loss of weight	Biliary obstruction	Biliary sphincterotomy and laparotomy	Alive
7	Chogle et al. [24]	3-year-old female	Cramping abdominal pain, vomiting, loss of weight	Biliary obstruction causing cholestasis	Laparotomy	Alive
8	Kim et al. [25]	75-year-old female	epigastric pain, nausea and vomiting	Acute pancreatitis and subsequent biliary obstruction	Laparotomy	Alive
9	Shawis et al. [26]	14-year-old female	Severe abdominal pain, bilious vomiting, bloody stool	Transient pancreatitis	Laparotomy	Alive
10	Vellaisamy et al.	37-year-old female	Acute exacerbation of abdominal pain radiating to the back, fever, halitosis, early satiety	Pancreatitis and acute cholangitis	Laparotomy	Alive

## 3 Discussion

Trichobezoars are conglomerations of hair that accumulate in the gastrointestinal tract. Trichobezoars are usually present in the stomach. Rarely, there might be an extension into the small intestine called Rapunzel syndrome presenting with intestinal obstruction, as seen in our case [1]. First reported in 1968, there have been less than 110 cases reported all over the world as per a recent review [5]. The majority of the cases are seen in patients younger than 20 years, with one of the largest case series reporting an average age of 5–23 years, therefore this case a relatively late presentation at 37 years [6].

This condition remains asymptomatic for a long time with symptoms appearing only in the late stages. Symptoms are very vague, such as abdominal pain (46.66%), nausea and vomiting (44.44%), obstruction (20%), abdominal distension (8.88%) and weight loss (8.88%) [7]. The clinical and radiological features help us narrow the diagnosis (Table 2).

**Table 2:** Clinical and radiological differential diagnoses

Differential diagnosis	Clinical findings	Radiologic findings
Phytobezoars	Non-specific, loss of appetite, nausea, vomiting; examination shows epigastric mass, relevant history	Intraluminal mass with air bubbles and site of the obstruction on CT
Gastrointestinal stromal tumour	Silent until large. Nonspecific symptoms – abdominal pain, fatigue, dyspepsia, nausea, anorexia, weight loss, fever and obstruction.	Exophytic mass arising from wall of hollow viscus, with ulceration and necrosis confirmed by CT
Choledocholithiasis	Biliary colic, jaundice, fever, nausea, vomiting, loss of appetite, pain radiating to back (pancreatitis), Murphy sign positive	USG showing opacity and dilated bile ducts, CECT shows central density and surrounding attenuation
Gastric carcinoma	Pain, nausea, vomiting due to outlet obstruction and early satiety. Examination may show visible gastric peristalsis	CT shows polypoid mass with ulceration, focal thicken of wall and mucosal irregularity
Trichobezoar	Nausea, pain, vomiting with or without complications of perforation, pancreatitis, cholangitis	CT scan shows intraluminal mottled mass of mesh-like appearance in the epigastrium (can extend in Rapunzel Syndrome)
Internal hernia	Vague epigastric colicky pain, nausea, vomiting, constipation, abdominal distension	CT scan shows encapsulation of bowel loops, obstruction presenting as dilation and stasis

Majority of the patients have a psychiatric history of trichotillomania, trichophagia or pica (consumption of non-nutritive substances like ice, usually accompanied by iron deficiency anaemia) [8]. It is estimated that 1 in 2,000 children suffer from trichotillomania, but it is severely underdiagnosed [8]. However, this history is very difficult to elicit upon initial presentation unless it is specifically asked for ref. [9]. Therefore, in female patients with no other apparent causes of pain, a history of these psychiatric illness plays a major role in the diagnosis. In cases where the history is not apparent, careful examination of the scalp for patchy alopecia might aid in the diagnosis.

In a very small number of cases, it goes undetected for a long period of time causing biliary or pancreatic obstruction, gastric perforation or intussusception. Pancreatitis due to obstruction of Ampulla of Vater by the bezoar has been reported in only four cases of Rapunzel syndrome thus far ref. [10]. Irritation by the bezoar tail extending into the duodenum causes oedema and obstruction of the drainage of bile which was first reported by Schreiber et al. [11]. Derangement of liver enzymes, acute cholangitis and cholestasis are some of the rare biliary complications caused by the bezoar [11]. Based on a review of over 20 years – this is the only case of Rapunzel syndrome in a woman, with complications of both acute cholangitis and pancreatitis. Severe malnutrition and enteropathy are also seen in some cases as the mass prevents the absorption of nutrients in the stomach and small intestine [12].

Ultrasound may show an echogenic mass, but a CT scan is the diagnostic tool of choice since it provides a clear image of the bezoar delineated by the contrast dye, as seen in our case (Figure 1) [13]. Endoscopy also shows a large concretion of hair which is putrid and foul-smelling due to decomposition and fermentation of fats.

Treatment depends on size and location. Successful management involves complete removal as well as ensuring the prevention of recurrence. Previous literature shows that only small bezoars can be successfully removed by endoscopy. There is still a risk of esophagitis and perforation, which is higher with large bezoars [14]. For giant bezoars with invasion into the duodenum, gastrotomy is the first-line treatment as it reports the least complications, recurrence and also allows screening for satellite lesions [15,16]. Due to the large size of the bezoar and the complications involved, endoscopic removal posed a higher risk as it was nearly impossible to fragment and remove the entire bezoar successfully. Intraoperatively, we noticed the bezoar tail obstructing the Ampulla of Vater, confirming our suspected diagnosis. Some cases of gastric trichobezoar can be managed laparoscopically as stated by Yau et al. as it allows for a shorter postoperative stay and a smaller scar, yet the risk of intrabdominal spillage and incomplete removal are concerns [16,17]. In the largest review of the management of trichobezoars, 100 of 108 patients (92.5%) were treated by laparotomy, with only a 12% complication rate making it the treatment of choice for trichobezoars especially in Rapunzel syndrome [15]. The main goal is to prevent recurrence and hence proper psychiatric counselling and follow-up is of utmost importance, as highlighted by our case. Although malpractice claims in India are far fewer than those in the United States and other Western countries, they are on the rise with the recent addition of high compensation awards for negligence [18]. With this background, a detailed written informed consent was obtained from the patient before surgery, as well as before the submission of the case report after all advantages and disadvantages were clearly described. However, to date, there have been no reports of medicolegal cases with diagnoses of Rapunzel syndrome.

The case reveals that Rapunzel syndrome can have varied, often non-specific presentations as well as a myriad of complications such as cholangitis and pancreatitis, and it must be high on the differential diagnosis for patients with a psychiatric history.

## 4 Conclusions

Rapunzel syndrome is a very rare condition usually seen in young females with trichotillomania and trichophagia. Hence, we report a rare case of Rapunzel syndrome presenting with both acute cholangitis and pancreatitis. A history of psychiatric illness, a thorough clinical examination and confirmation of the diagnosis using investigations is key to making an accurate diagnosis. Surgical removal is the first line treatment for giant bezoars and is associated with the least complications. Hence, when a female patient with a history of psychiatric illness presents with features of intestinal obstruction, a high clinical suspicion must be held for trichobezoar, and regular post-operative psychiatric counselling is of utmost importance to prevent a recurrence.
